# Flexible Splinting: A Minimally Invasive Approach to Managing a Pediatric Mandibular Fracture With Traumatic Bone Loss

**DOI:** 10.7759/cureus.87098

**Published:** 2025-07-01

**Authors:** Fasna K, Mahendra Kumar Jindal, Mohammad Atif, Saima Y Khan

**Affiliations:** 1 Department of Pediatric and Preventive Dentistry, Dr. Ziauddin Ahmad Dental College, Aligarh Muslim University, Aligarh, IND

**Keywords:** composite and wire, flexible splinting, minimally invasive approach, pediatric mandibular fracture, traumatic bone loss

## Abstract

Pediatric mandibular fractures necessitate a specialized treatment approach due to the complexities of craniofacial development, dentition, and active bony growth centers. This case report describes the management of an eight-year-old boy who sustained a mandibular fracture with the traumatic loss of a triangular bone fragment following a fall. Clinical and radiographic evaluation confirmed a compound comminuted fracture of the mandibular body. Given the minimal segmental mobility and intact occlusion, a conservative approach using flexible splinting with composite and braided stainless steel wire was adopted. The patient showed successful healing with complete bone formation at the fracture site after 12 months of follow-up. This case underscores the effectiveness of a minimally invasive approach for managing pediatric mandibular fractures, with the added benefits of preserving growth potential and minimizing complications.

## Introduction

The impulsive and adventurous behavior commonly observed in children often results in their participation in physical activities without adequate consideration of consequences or adherence to parental guidance. However, children tend to experience fewer facial injuries than adults due to the more elastic nature of growing bones, a dense layer of adipose tissue, a greater cancellous-to-cortical bone ratio, and pliable suture lines. Additionally, the presence of developing tooth buds and the absence of sinus pneumatization contribute to the enhanced stability of the facial skeleton in children [1]. Studies have reported that mandibular fractures account for 24%-44% of all pediatric facial fractures, making them one of the most commonly encountered fractures in growing individuals [2]. According to a multicentric study by Segura-Palleres et al., the most frequent causes of maxillofacial fractures in children were road traffic accidents (36%), followed by falls (24%) and sports injuries (21%) [3].

The treatment of mandibular fractures in children requires a specialized approach due to distinct anatomical and developmental factors. Unlike adults, where absolute reduction and rigid fixation are often necessary, pediatric cases demand minimal skeletal manipulation to preserve the growth potential. This distinction is driven by ongoing mandibular growth, dentition development, and the presence of active bony growth centers. Additionally, the smaller jaw size, close proximity of developing permanent tooth buds to the mental and mandibular nerves, and the dense arrangement of deciduous teeth present unique challenges. These factors not only increase the complexity of pediatric mandibular fracture management but also elevate the likelihood of developmental disruptions and persistent functional complications [1]. Therefore, conservative methods are preferred in pediatric mandibular fracture management, as they preserve the periosteal sleeve, maintain soft tissues, and reduce psychological trauma. Additionally, conservative approaches are generally associated with a lower risk of temporomandibular joint ankylosis; however, in cases involving condylar fractures, missed diagnosis or prolonged immobilization may predispose to fibrosis or bony ankylosis due to the joint's high osteogenic potential [4]. Surgical intervention should be reserved for cases where it is absolutely necessary [5].

This case report details the management of mandibular fracture in an eight-year-old boy with traumatic loss of a triangular bone fragment at the left lower border of the mandible. The fracture was treated using flexible splinting with wire and composite, resulting in successful healing without complications such as non-union or malunion.

## Case presentation

**Figure 1:**
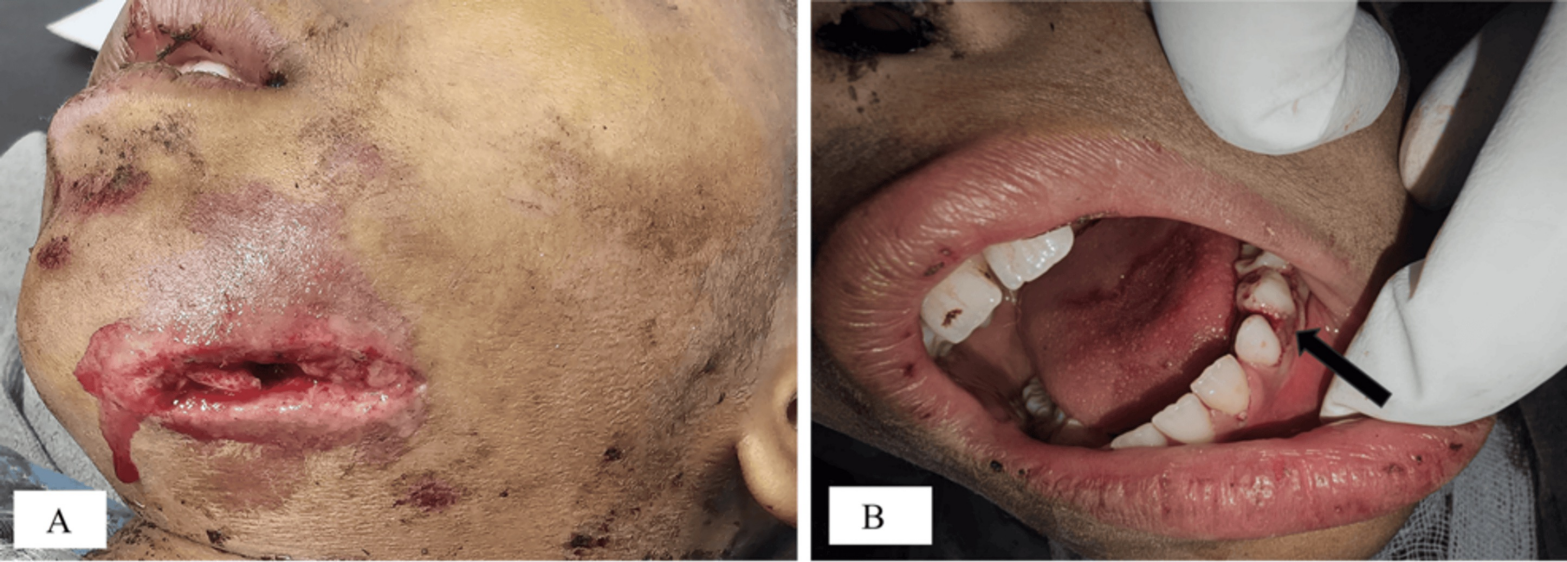
Preoperative photographs: (A) extraoral; (B) intraoral

An eight-year-old boy presented to the emergency trauma unit of a tertiary care center with a chief complaint of mouth and chin injuries sustained 3-4 hours earlier following a self-induced fall into a well approximately 22 feet deep. A comprehensive patient history revealed no loss of consciousness, vomiting, convulsions, or bleeding from the ears or nose. The patient was moderately built and nourished with no relevant past medical or dental history. Clinical examination revealed a fractured left hand, which was managed by the Department of Orthopedics. Extra-orally, a 5 cm deep laceration was noted along the left lower facial border with profuse bleeding (Figure 1A).

On palpation, a triangular bone fragment was detected alongside the damaged tissue within the laceration. Bilateral temporomandibular joint function was intact, with no signs suggestive of condylar fracture. Intraoral examination revealed segmental mobility between the left lower primary canine and primary first molar, accompanied by a slight step deformity causing minor occlusal misalignment. The patient was in the mixed dentition stage, with no other significant findings noted (Figure 1B).

**Figure 2:**
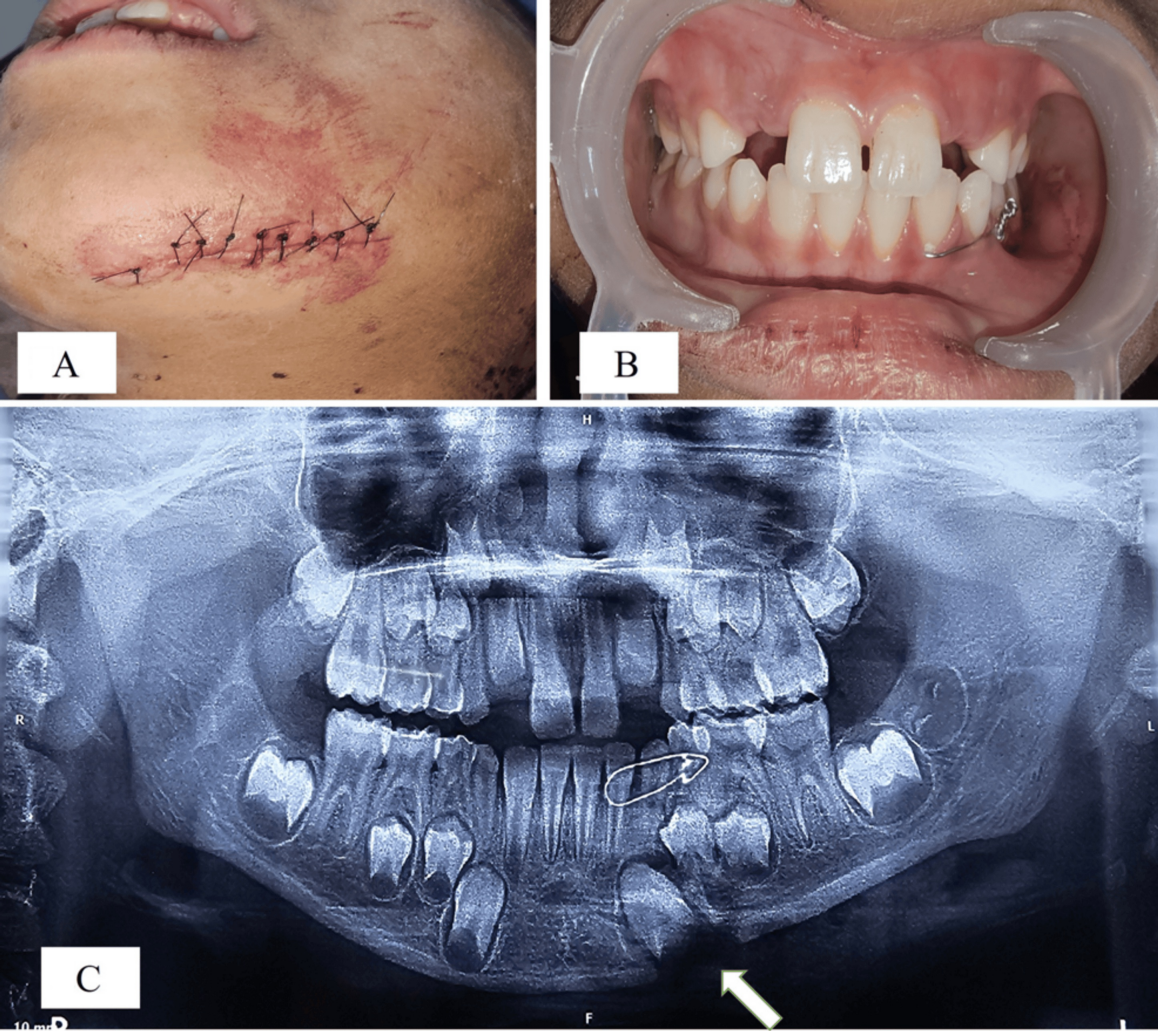
(A) Double-layered suturing with Vicryl 4-0 and Nylon 4-0; (B) intraoperative clinical photograph showing the proper occlusion after bridle wiring; (C) orthopantomogram (OPG) showing the bridle wiring and lost triangular bone fragment near tooth bud 33

After obtaining neural clearance and informed consent from the patient, a 1 cm wedge-shaped bone fragment, found among the damaged tissue, was removed as it was completely detached from the injury site. After cleaning the wound, hemostasis was achieved through pressure application with sterile cotton, followed by suturing of the laceration by double-layered suturing with Vicryl 4-0 (Ethicon, Inc., Somerville, MA, US) and Nylon 4-0 (Ethicon, Inc.) under local anesthesia at the trauma center (Figure 2A). Bridle wiring was placed from tooth 32 to 74 using a 26-gauge wire to temporarily stabilize the fractured segment. Satisfactory occlusion was achieved through the bridle wiring alone (Figure 2B). The patient was prescribed an oral antibiotic (syrup Augmentin^®^ containing amoxicillin 200 mg and clavulanic acid 28.5 mg per 5 mL), 5 mL three times daily for five days, along with an analgesic (syrup Ibugesic Plus^®^ containing ibuprofen 100 mg and paracetamol 162.5 mg per 5 mL), also 5 mL three times daily for five days. The patient was then referred to the Department of Pediatric and Preventive Dentistry for detailed radiographic evaluation and definitive management of the fracture, as per the institutional protocol, wherein all patients under 14 years of age presenting with maxillofacial trauma are managed by this department.

The patient reported to the Outpatient Department (OPD) on the following day. An orthopantomogram (OPG) was advised using the Carestream CS 8000 panoramic imaging system (68-76 kV, 8-10 mA, exposure time: 11 seconds) (Carestream Dental LLC, Atlanta, GA, US), which confirmed a compound comminuted fracture of the mandibular body. The fracture line extended between teeth 73 and 74, with traumatic loss of a small bone fragment from the lower border of the mandible near the tooth bud of tooth 33 (Figure 2C).

Considering the minimal segmental mobility and intact occlusion after the bridle wiring procedure performed a day before, a conservative treatment approach was selected, focusing on fracture stabilization through flexible splinting with composite and braided stainless steel wire.

**Figure 3:**
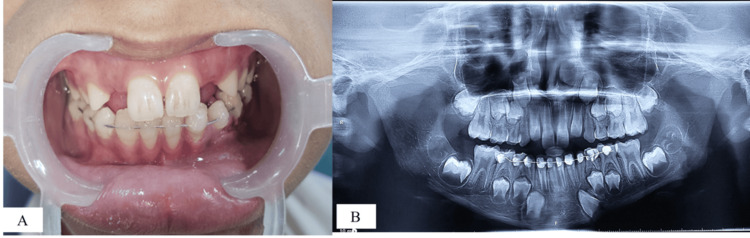
Stabilization using flexible splinting: (A) immediate postoperative clinical photograph; (B) immediate postoperative OPG after flexible splinting OPG: orthopantomogram

Splinting was performed from the second primary molar in the fourth quadrant to the contralateral side. A precurved 0.4 mm braided stainless steel wire of appropriate length was adapted to the lower arch. Following isolation, the enamel was etched with 37% phosphoric acid (Ivoclar Vivadent, Eco-Etch^®^, Schaan, Liechtenstein) for 30 seconds, then rinsed and dried. An adhesive agent (Te-Econom Bond^®^, Ivoclar Vivadent) was applied to the etched enamel surfaces in accordance with the manufacturer’s instructions. The preadapted stainless steel wire was then secured using a light-cured composite resin (Te-Econom Plus^®^, Ivoclar Vivadent) and polymerized for 20 seconds with an LED curing unit (SmartLite^®^ Focus^®^, Dentsply Sirona, Charlotte, NC, US), ensuring proper occlusal alignment (Figures 3A, 3B).

**Figure 4:**
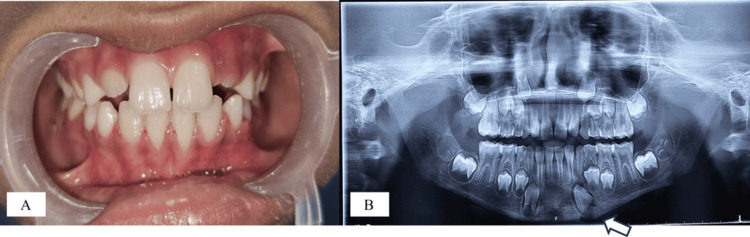
Two-month follow-up pictures: (A) clinical photograph after splint removal showing proper occlusion; (B) OPG after splint removal showing the initiation of calcification at the lost bone fragment site OPG: orthopantomogram

The patient was advised to maintain good oral hygiene, adhere to a soft diet for the duration of the splint retention period, and continue the prescribed medications as directed. The sutures were removed after one week, following satisfactory healing at the laceration site. The splint was kept in place for two months to allow for the initiation of bone calcification at the fracture site. The patient was regularly seen for follow-up visits during this period. After two months, the OPG showed signs of bone calcification and the patient remained asymptomatic, with no segmental mobility. As a result, the splint was removed (Figures 4A, 4B).

**Figure 5:**
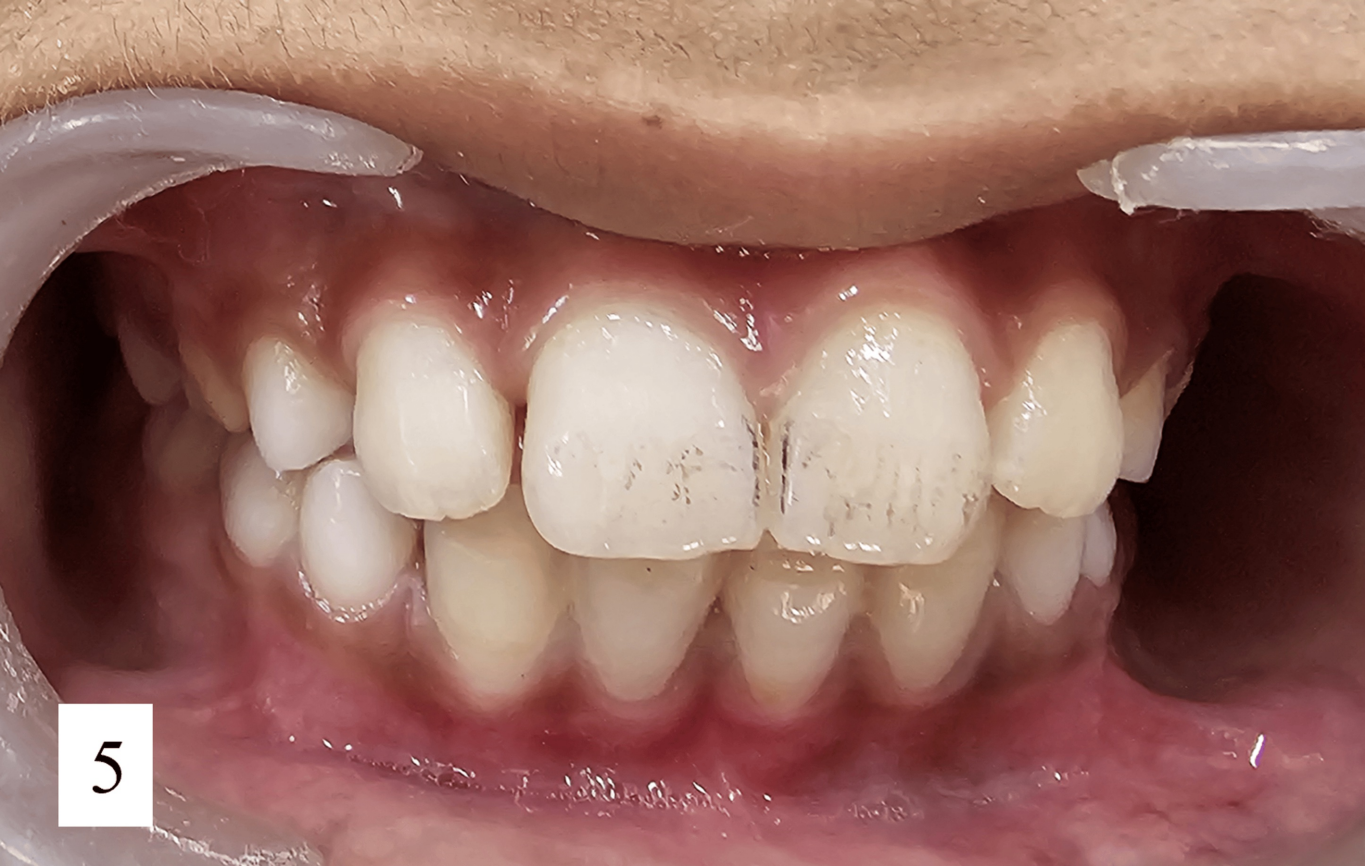
Clinical photograph after 6 months showing satisfactory healing and proper occlusion

Figure 5 presents a clinical photograph taken after six months, demonstrating satisfactory healing and proper occlusion with positive patient feedback.

**Figure 6:**
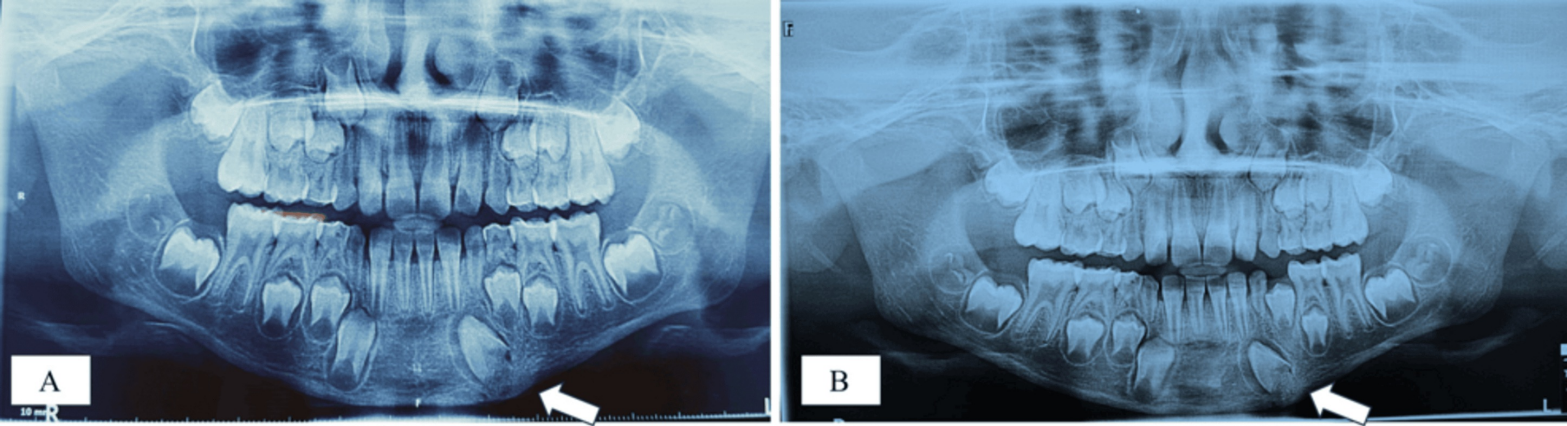
Follow-up OPGs; (A) OPG after 6 months showing complete bone formation at the lost bone fragment site; (B) OPG after 1 year revealing that the left canine bud remained in the same position without any signs of root development OPG: orthopantomogram

The OPG taken after six months showed complete bone formation at the site of the lost bone fragment (Figure 6A). A follow-up OPG after 12 months was performed to evaluate the status of the left permanent canine bud, which revealed that it remained in the same position without any signs of root development (Figure 6B), suggesting a high likelihood of ankylosis followed by replacement resorption.

## Discussion

The healing capacity in children is enhanced due to their high physiological activity and the robust osteoregenerative potential of the periosteum. Bone healing occurs more rapidly in pediatric patients, with osteogenesis typically initiating by the fourth week in individuals under 18 years of age, whereas in adults, it generally begins around the eighth week. Consequently, any delay in treatment poses a greater concern in pediatric patients compared to adults [6].

Mandibular fractures can be categorized as favorable or unfavorable based on the muscular forces and the potential displacement of the fracture segments. When the muscular forces are strong enough, closed reduction techniques may become ineffective if the segments cannot be properly aligned and stabilized against the muscular pull [7]. Therefore, the type of pediatric mandibular fracture plays a crucial role in determining the appropriate treatment approach, ranging from minimally invasive techniques such as closed reduction using acrylic splints, circumferential wiring, arch bars, or gunning splints, to more invasive procedures like open reduction with internal rigid fixation for effective stabilization [5].

Greenstick fractures and minimally displaced mandibular fractures can typically be treated using minimally invasive techniques. However, for severely displaced mandibular fractures, open reduction methods are the standard of care for management. Intermaxillary fixation (IMF) can cause risks such as avulsion of primary teeth that lack adequate stability to endure the applied forces by IMF. The unique structure of primary teeth, with broad cervical margins and tapered occlusal surfaces, makes the application of IMF devices or eyelets particularly difficult. Additionally, these devices can hinder normal eating habits in children, leading to noticeable weight and protein loss, decreased lung capacity, and a heightened risk of gastric content aspiration. The wires used in IMF can also cause discomfort and may damage the surrounding periodontal tissues [1].

In the present case, flexible splinting using a wire-composite technique was selected as the treatment approach due to minimal displacement and limited mobility of the fractured mandibular segments. Despite the application of bridle wiring, the patient’s occlusion remained unaffected, further supporting the decision to adopt a conservative method of stabilization. This approach was particularly influenced by the need for prolonged yet minimally invasive stabilization to facilitate calcification at the site of the lost bone fragment. Moreover, given the patient’s young age and ongoing craniofacial development, preserving the integrity of the underlying developing tooth buds was of paramount importance. The flexible wire-composite splint was advantageous in this regard, as it exerted minimal pressure on the alveolar structures and avoided interference with erupting dentition. Additionally, the splint did not introduce any occlusal discrepancies, thereby allowing the patient to maintain a normal diet-a crucial factor considering the extended stabilization period required. Maintaining adequate nutrition during this time was essential for optimal healing, and the selected method effectively supported both biological and functional recovery. Several studies support the implementation of minimally invasive methods for the treatment of pediatric mandibular fractures. In a study by Kaban et al., 10 patients with non-displaced mandibular fractures were successfully treated using only Barton bandages, achieving uncomplicated bone healing [8]. Similarly, a retrospective study by Li et al. demonstrated the effective rehabilitation of six children with mandibular fractures using Quartz fiber splints [9].

Vacuum-formed splints have recently been introduced for managing pediatric mandibular fractures [10]; however, they were not considered suitable in this case. Although these splints accurately replicate occlusal morphology, they inherently create an interocclusal gap of approximately 2 mm, which can be uncomfortable and often poorly tolerated by pediatric patients. Furthermore, prolonged use may compromise masticatory efficiency and nutritional intake-an important consideration, especially in cases requiring extended stabilization to support bone healing and calcification. In contrast, flexible splints offer several advantages in pediatric cases. They eliminate the need for laboratory fabrication, allow for natural intercuspation, and ensure better patient compliance due to improved comfort and minimal interference with oral functions.

Managing wedge-shaped segments at the inferior border of the mandible presents a challenge, and the literature remains inconclusive on whether surgical realignment and stabilization of the segment are preferable or if leaving it unexposed is a better approach to preserve periosteal integrity and vascularization [11]. Heslop et al. [12] recommended realigning the bone segments if they remain connected to the muscle or periosteum, while advising their removal if detached or minute. Block et al. stated that fibrous connective tissue may surround the unaligned bone fragments, losing their blood supply and eventually undergoing necrosis [13]. Blinder and Taicher [11] reported that if the triangular fragment remains unexposed, it is best to keep it undisturbed and unmanipulated to maintain vascularization and reduce the risk of infection. In the present case, the bone fragment was relatively small and completely detached from the fracture site without any periosteal attachment. Therefore, its removal was preferred, along with minimal stabilization of the fracture site, which yielded satisfactory results.

In this case, the left permanent canine tooth bud was nearly exposed due to the traumatic loss of a bone fragment. However, during the initial trauma management, it was decided not to remove the tooth bud to prevent further damage to surrounding structures. Because a bone fragment has already been lost, removing the tooth bud would not only further weaken the mandible but also disrupt the healing process. The outcome of an unerupted tooth in the line of fracture can vary and may include eruption without complications, delayed eruption, displacement, impaction, or resorption. Koenig et al. [14] reported that 82% of tooth buds located in the fracture line exhibited normal eruption or a normal pre-eruption appearance on radiographs, while 18% remained unerupted and one tooth bud showed signs of resorption. According to Macan et al., tooth buds within the fracture line should be removed only in cases of avulsion or confirmed follicular disruption, as the follicle-being more elastic than surrounding tissue-helps protect the developing tooth, allowing most to erupt normally if the follicle remains intact [15]. In this case, even after one year of trauma, the left canine bud remained in the same position without any signs of root development, whereas the contralateral canine bud exhibited significant root development. This suggests that the canine bud located within the fracture line is more likely to undergo resorption. The future management plan involves periodic clinical and radiographic monitoring at six-month intervals. If the canine bud remains unerupted and ankylosis or resorption is confirmed, treatment will be based on the clinical presentation. No intervention will be necessary if the tooth remains asymptomatic and does not interfere with adjacent structures or occlusal development. However, if any pathology arises, surgical removal may be considered. Orthodontic space management followed by prosthetic rehabilitation, such as a resin-bonded bridge or dental implant after skeletal maturity, may also be planned depending on the patient's growth and occlusal status.

The limitation of this case report was the unavailability of 3D imaging, such as cone-beam computed tomography (CBCT). Additionally, definitive treatment could not be provided at the trauma center on the first day of the patient's visit due to limited facilities.

## Conclusions

In this case, a composite flexible splint was successfully used to manage a favorable mandibular fracture with associated traumatic bone loss in a pediatric patient, resulting in satisfactory clinical outcomes. While current evidence is limited, and definitive conclusions regarding its universal applicability cannot be drawn, this technique shows promise. Given its minimally invasive nature and ease of application, composite flexible splinting may serve as a viable alternative for managing selected cases of pediatric mandibular fractures. Further clinical studies are essential to validate the long-term outcomes and standardize the indications for this technique in pediatric populations.

## References

[1] Aizenbud D, Hazan-Molina H, Emodi O, Rachmiel A (2009). The management of mandibular body fractures in young children. Dent Traumatol.

[2] Cleveland CN, Kelly A, DeGiovanni J, Ong AA, Carr MM (2021). Maxillofacial trauma in children: association between age and mandibular fracture site. Am J Otolaryngol.

[3] Segura-Palleres I, Sobrero F, Roccia F (2022). Characteristics and age-related injury patterns of maxillofacial fractures in children and adolescents: a multicentric and prospective study. Dent Traumatol.

[4] Bottini GB, Roccia F, Sobrero F (2024). Management of pediatric mandibular condyle fractures: a literature review. J Clin Med.

[5] Bansal A, Yadav P, Bhutia O, Roychoudhury A, Bhalla AS (2021). Comparison of outcome of open reduction and internal fixation versus closed treatment in pediatric mandible fractures-a retrospective study. J Craniomaxillofac Surg.

[6] Kawai T, Murakami S, Hiranuma H, Sakuda M (1997). Radiographic changes during bone healing after mandibular fractures. Br J Oral Maxillofac Surg.

[7] Blitz M, Notarnicola K (2009). Closed reduction of the mandibular fracture. Atlas Oral Maxillofac Surg Clin North Am.

[8] Kaban LB, Mulliken JB, Murray JE (1977). Facial fractures in children: an analysis of 122 fractures in 109 patients. Plast Reconstr Surg.

[9] Li L, Acharya K, Ghimire B (2023). Conservative management of mandibular fractures in pediatric patients during the growing phase with splint fiber and ligature arch wire. BMC Oral Health.

[10] Nilesh K, Mahamuni A, Taur S, Vande AV (2020). A simple novel technique for the management of a dentoalveolar fracture in a pediatric patient using a vacuum-formed splint. J Dent Res Dent Clin Dent Prospects.

[11] Blinder D, Taicher S (1995). Management of triangular fragments of the inferior border of the mandible. Oral Surg Oral Med Oral Pathol Oral Radiol Endod.

[12] Heslop IH, Clarke PB, Becker R (1985). Mandibular fractures: treatment by open reduction and direct skeletal fixation. Maxillofacial Injuries.

[13] Block MS, Provenzano J, Neary JP (1990). Complications of mandibular fractures. Oral Maxillofacial Surg Clin North Am.

[14] Koenig WR, Olsson AB, Pensler JM (1994). The fate of developing teeth in facial trauma: tooth buds in the line of mandibular fractures in children. Ann Plast Surg.

[15] Macan D, Brajdic D, Zajc I (2012). The fate of teeth in mandibular fracture line. Rad.

